# Chronic Lead Exposure in Adult Mice: Associations with miR-671/CDR1as Regulation, NF-κB Signaling, and Alzheimer’s Disease-like Pathology

**DOI:** 10.3390/toxics12060410

**Published:** 2024-06-04

**Authors:** Mengyun Qiao, Haitao Yang, Li Liu, Tao Yu, Haihua Wang, Xiao Chen, Yi Zhang, Airu Duan, Shujun Lyu, Siyu Wu, Jingwei Xiao, Bin Li

**Affiliations:** 1 State Key Laboratory of Trauma and Chemical Poisoning, National Institute for Occupational Health and Poison Control, Chinese Center for Disease Control and Prevention, Beijing 100050, China; 2 Department of Toxicology, National Institute for Occupational Health and Poison Control, Chinese Center for Disease Control and Prevention, Beijing 100050, China; 3 Key Laboratory of Chemical Safety and Health, Chinese Center for Disease Control and Prevention, Beijing 100050, China; 4 Key Laboratory of Environmental Medicine and Engineering, Ministry of Education, School of Public Health, Southeast University, Nanjing 210009, China

**Keywords:** lead, Alzheimer’s disease, learning memory, CDR1as, miR-671, NF-κB

## Abstract

Long-term exposure to lead (Pb) can result in chronic damage to the body through accumulation in the central nervous system (CNS) leading to neurodegenerative diseases, such as Alzheimer’s disease (AD). This study delves into the intricate role of miR-671/CDR1as regulation in the etiology of AD-like lesions triggered by chronic Pb exposure in adult mice. To emulate the chronic effects of Pb, we established a rodent model spanning 10 months of controlled Pb administration, dividing 52 C57BL/6J mice into groups receiving varying concentrations of Pb (1, 2, or 4 g/L) alongside an unexposed control. Blood Pb levels were monitored using serum samples to ensure accurate dosing and to correlate with observed toxicological outcomes. Utilizing the Morris water maze, a robust behavioral assay for assessing cognitive functions, we documented a dose-dependent decline in learning and memory capabilities among the Pb-exposed mice. Histopathological examination of the hippocampal tissue revealed tell-tale signs of AD-like neurodegeneration, characterized by the accumulation of amyloid plaques and neurofibrillary tangles. At the molecular level, a significant upregulation of AD-associated genes, namely amyloid precursor protein (APP), β-secretase 1 (BACE1), and tau, was observed in the hippocampal tissue of Pb-exposed mice. This was accompanied by a corresponding surge in the protein levels of APP, BACE1, amyloid-β (Aβ), and phosphorylated tau (p-tau), further implicating Pb in the dysregulation of these key AD markers. The expression of CDR1as, a long non-coding RNA implicated in AD pathogenesis, was found to be suppressed in Pb-exposed mice. This observation suggests a potential mechanistic link between Pb-induced neurotoxicity and the dysregulation of the CDR1as/miR-671 axis, which warrants further investigation. Moreover, our study identified a dose-dependent alteration in the intracellular and extracellular levels of the transcription factor nuclear factor-kappa B (NF-κB). This finding implicates Pb in the modulation of NF-κB signaling, a pathway that plays a pivotal role in neuroinflammation and neurodegeneration. In conclusion, our findings underscored the deleterious effects of Pb exposure on the CNS, leading to the development of AD-like pathology. The observed modulation of NF-κB signaling and miR-671/CDR1as regulation provides a plausible mechanistic framework for understanding the neurotoxic effects of Pb and its potential contribution to AD pathogenesis.

## 1. Introduction

Lead (Pb) is a common industrial toxicant and widely distributed environmental pollutant [1]. With the development of society and the needs of the industrial revolution, the demand for Pb has increased, and the exposure of occupational groups to Pb in the workplace has become a common health concern in countries around the world [2]. In 2010, Pb has been listed by the World Health Organization (WHO) as one of the top ten environmental pollutants harmful to human health in the world [3].

Pb can accumulate in the human body and cause damage to multiple organ systems, including the nervous system, digestive system, respiratory system, etc., especially the central nervous system (CNS) [4]. In recent years, increasing attention has been paid to the role of chronic lead exposure in the development of neurodegenerative diseases, such as Alzheimer’s disease (AD). It has been reported that there is no safe threshold for lead exposure, and even low-dose exposure can induce irreversible brain damage, such as neuronal apoptosis and loss of learning and memory capacity [5]. Children and pregnant women are the most liable to suffer lead poisoning. Pb exposure during early life causes more severe neurological life-long problems [6,7], later leading to the development of AD. Most of the current studies have mainly focused on the mechanisms related to neurological damage caused by lead exposure in early life while neglecting to explore the mechanisms of AD caused by lead exposure in adulthood.

Much epidemiologic and experimental research evidence suggests that Pb exposure can lead to the development of AD [8,9], but the associated mechanistic pathways have not been elucidated. Mammalian genes are controlled by several genetic and epigenetic mechanisms, and circular RNAs (circRNAs) have been identified as vital arbiters of gene expression. As novel noncoding RNAs, circRNAs, unlike linear RNAs, can form a covalently closed continuous loop with their 3′ heads and 5′ tails bound together [10]. CircRNAs have been detected in multiple organisms; their unique closed-loop structure makes them more resistant to digestion by RNAse R, and they are ideal biomarkers for diagnosis [11]. In recent years, tens of thousands of circRNAs have been identified through deep sequencing and bioinformatic analysis, and their biological functions have begun to be explored [12]. Among them, CDR1as has been widely studied as an important circRNA. CDR1as can act as a “molecular sponge” for microRNAs (miRs) or can directly regulate transcription and translation processes, and the proper functioning of these roles is an important basis for maintaining neurological homeostasis [13,14]. The development of AD is closely related to the dysregulation of CDR1as expression in the brain [15,16,17,18]. In contrast, the reduced expression level of CDR1as in the brains of AD patients may be due to the fact that microRNA miR-671 has a binding site that is almost completely complementary to CDR1as and induces cleavage of CDR1as by Argonaute 2 (AGO2) protein, which may be one of the important pathways for the reduced expression level of CDR1as in the brains of AD patients [17,19]. However, the neurobiological functions of CDR1as as a risk factor for environmental risk factor exposure conditions have not been elucidated.

Dysregulation of CDR1as regulatory expression was found in vitro experimental studies to be associated with alterations in downstream related signaling pathways, such as the NF-κB signaling pathway [20]. NF-κB signaling pathway is a classical signal transduction pathway that plays a critical role in the transcriptional activation of genes associated with AD pathology. Previous studies have demonstrated that NF-κB can facilitate the expression of key AD-related genes such as beta-site APP-cleaving enzyme 1 (BACE1) and amyloid precursor protein (APP) by binding to specific promoter regions [21,22,23]. This increased expression of APP and BACE1 can promote amyloid beta (Aβ) production and tau protein hyperphosphorylation, which are believed to be a major contributor to the development of AD [24].

There is a lack of studies on the mechanisms associated with Pb exposure resulting in AD in adults, especially the lack of in vivo experimental studies under long-term poisoning conditions. Few studies have reported on the effects of Pb exposure on the regulatory expression of CDR1as. Therefore, we established a mouse model of AD in which Pb exposure begins in adulthood and examined the expression differences of several key CDR1as-associated factors, miR-671, AGO2, CDR1as, NF-κB, BACE1, APP, Aβ, and p-tau, under different concentrations of Pb exposure conditions to preliminarily explore the role of miR-671/CDR1as regulation in the effect of Pb exposure on Aβ and p-tau production. Our findings provide a basis for the mechanism of Pb exposure causing AD and also provide an important theoretical and experimental foundation for further revealing not only the specific evolutionary mechanism of AD caused by Pb exposure but also the potential intervention targets for later therapeutic development.

## 2. Materials and Methods

### 2.1. Animals and Experimental Design

Animal experiments were approved by the Laboratory Animal Welfare and Ethics Committee of the National Institute for Occupational Health and Poison Control, Chinese Center for Disease Control and Prevention (No. EAWE-2021-09) and complied with the Institute Animal Ethics Guidelines. Specific pathogen-free C57BL/6J mice (6–8 w, 20–30 g, male) were purchased from Beijing Sibeifu Biotechnology Co., Ltd. (Beijing, China) (No. SCXK(Jing)2019-0010). All mice were given free access to standard diet and water and were housed with temperature (18–25 °C) and humidity (40–70%) control and with a light-dark cycle of 12 h/12 h. After 1 week of acclimatization, 52 C57BL/6J mice were randomly assigned to four groups (n = 13 for each group): (a) control group, (b) 1 g/L–low-dose exposure group, (c) 2 g/L–medium-dose exposure group, and (d) 4 g/L–high-dose exposure group [25].

### 2.2. Chronic Pb Exposure

All the Pb-exposed mice were administered with lead-acetate trihydrate (L812611, Macklin, Shanghai, China) through drinking water that was provided ad libitum for 10 months, while the control mice received sterile deionized water(dH_2_O) (L25, Yangzhou Zhongken Foods Co., Ltd., Yangzhou, China). The Pb uptake in mice was estimated by measuring the water intake of animals every two days. Mice were weighed and recorded weekly.

### 2.3. Morris Water Maze

Initially, there was a visible platform phase; the platform was set 1 cm above the water level and marked with black tape so that the mice could locate the platform using a local visual stimulus rather than relying on spatial orientation to extra-maze cues. Mice were placed in the pool for 120 s the day before training to acclimatize to the pool environment. The next five days comprised the hidden platform phase; we added additional water to the pool to submerge the platform to 1 cm below the surface during the navigation test. The mice were placed smoothly into the water from the middle of the quadrant with their heads facing the wall of the pool. After the mouse was released, we retreated away from the pool to a constant position within the room, serving as an additional distal visual cue. The mice were permitted a maximum time of 120 s to find the platform and 10 s to stay on the hidden platform, starting from their release in a randomly chosen quadrant. Mice failing to relocate the platform within 120 s were guided to the platform and allowed to stay on the platform for 10 s. On each testing day, the animals performed 4 trials separated by 30 min intervals. The mean avoidance latency was the average of the results of four training sessions at the end of each day’s training as the final performance of the mice. After the navigation test, the platform was removed, and a probe trial was performed to assess the memory abilities of the mice. Mice were permitted to swim freely for 120 s. The number of times that the mice crossed the location of the platform, and the first escape attempt latency were recorded. All data were video-recorded and computer-analyzed (EthoVision 13.0, Noldus, Wageningen, The Netherlands).

### 2.4. Blood and Hippocampal Tissue Sample Preparation

The mice were anesthetized with 2% sodium pentobarbital and 0.5 mL of abdominal aortic blood was collected and combined with heparin to prevent coagulation. The mice were subsequently decapitated and intact brains were stripped of dura mater on ice and weighed. Hippocampal tissues were quickly dissected, flash-frozen in liquid nitrogen, and stored at −80 °C for testing.

### 2.5. Estimation of Pb in Blood and Hippocampus

The Pb^2+^ content in the blood and hippocampus was measured using High Resolution Inductively Coupled Plasma Mass Spectroscopy (ICP-MS) [26]. Hippocampus samples were weighed and digested in concentrated nitric acid (0.5 mL) and heated in boiling water for 2 h. After cooling, the sample was washed three times and was dissolved in dH_2_O (9.8 mL). The whole blood sample (0.1 mL) was dissolved in 0.5% nitric acid solution (9.8 mL). The blood Pb and hippocampal Pb levels were determined by adjusting the instrument to the optimal operating condition according to the operating procedures of the ICP-MS instrument (PerkinElmer, Waltham, MA, USA).

### 2.6. Hematoxylin and Eosin (HE) Staining

The mice were anesthetized with 2% sodium pentobarbital. The left ventricle of the mice was cannulated and perfused with 4% paraformaldehyde, followed by dura mater stripping the intact brains. After rapid rinsing with normal saline (0.9%), brain tissues were fixed with 4% paraformaldehyde for 24 h. Then, the brain tissues were made into 4-μm-thick paraffin coronal sections by dehydration and embedding and HE staining was performed to observe the histopathological changes in the hippocampus of the mouse brain. Morphological changes in hippocampal structure were assessed by a professional pathologist.

### 2.7. Immunofluorescence Staining

Paraffin sections (4 μm) were incubated with rabbit monoclonal anti-Aβ IgG (1:250; ab201060, Abcam, Cambridge, UK) and rabbit monoclonal anti-p-tau IgG (1:250; ab254409, Abcam) overnight at 4 °C. Sections were then incubated with fluorescein isothiocyanate-conjugated goat anti-rabbit IgG (1:200; ab6721, Abcam) secondary antibody incubated for 2 h at room temperature. Nuclei were counterstained by incubation in 1 μg/mL 4′,6-diamidino-2-phenylindole (DAPI) for 5 min, followed by extensive washing in distilled water. Images were acquired using a confocal laser scanning microscope (Pannoramic MIDI, 3D HISTECH, Budapest, Hungary).

### 2.8. Quantitative Real-Time PCR (qRT-PCR)

Total RNA was extracted from hippocampal tissue samples using the Total RNA Extraction Kit (GPQ1801, GenePool, Beijing, China). RNA was electrophoresed on 1% agarose gel to verify RNA integrity. The mRNA was reverse transcribed to cDNA using the mRNA cDNA Synthesis Kit (GPQ1803, GenePool, Beijing, China) and then amplified using the mRNA/lncRNA qPCR kit (GPQ1808, GenePool, Beijing, China) in a LineGene 9600 Plus system (Bioer Technology, Hangzhou, China). The miRNA was reverse transcribed to cDNA using the miRNA cDNA Synthesis Kit (GPQ1804, GenePool, Beijing, China), which incorporates a poly(A) tailing step and then amplified using the miRNA qPCR Kit (GPQ1809, GenePool, Beijing, China) in a LineGene 9600 Plus system (Bioer Technology, Hangzhou, China). The primer sequences used are shown in Table 1, with GAPDH, β-actin, and U6 used as internal reference controls.

### 2.9. Western Blot

The hippocampal tissue was homogenized and lysed, and the total protein of the samples was extracted according to the instructions of the Protein Extraction Kit (GPP1815, GenePool, Beijing, China). The volume of each protein sample was adjusted to provide equal protein concentrations. SDS-PAGE electrophoresis was performed on a 12% separation gel with a 5% concentration gel at 80 V and 120 V for 1 h each. Gel bands were transferred to polyvinylidene difluoride membrane (PVDF) under 300 mA for 2 h. After transfer, the PVDF membranes were immersed in BSA blocking buffer and gently shaken on a shaker for 1 h. PVDF membranes were then placed in a hybridization bag and a primary antibody diluted with 1% BSA was added: rabbit monoclonal anti-NF-κB (1:1500; ab16502, Abcam), rabbit monoclonal anti-BACE1 (1:1000; ab183612, Abcam), rabbit monoclonal anti-APP (1:1000; ab32136, Abcam), rabbit monoclonal anti-Aβ (1:800; ab201060, Abcam), mouse monoclonal anti-tau (1:2000; ab254150, Abcam), rabbit monoclonal anti-p-tau (1:1000; ab254409, Abcam) and β-actin(1:3000; ab8226, Abcam). Bags were then sealed with a film sealer and incubated overnight at 4 °C. After incubation, the membrane was washed 3 times with TBST (5 min each time). The second antibody was diluted [horseradish peroxidase-labeled goat anti-rabbit IgG (1:5000; ab6721, Abcam), rabbit anti-lamin B1 IgG (1:1000; ab133741, Abcam)] and shaken gently at room temperature for 50 min, followed by washing 4 times with TBST (5 min each time). Finally, the PVDF membrane was immersed in ECL chromogenic solution for 1 min, exposed, developed, and fixed in a dark room. Grayscale values of the strips were imaged and analyzed with Quantity One v.4.6.2 software.

### 2.10. Nucleoplasmic Separation of Hippocampal Neuronal Cells

Nucleoplasmic separation of hippocampal neuronal cells was performed by manufacturer protocol using a Nucleoplasmic Separation Kit (P0028, Beyotime, Shanghai, China).

### 2.11. Statistical Analysis

SPSS26.0 software was used for statistical analysis. The continuous variables were expressed by Mean ± SEM. Prism GraphPad 9.0 and R 4.0.5 were used for plotting. The homogeneity for variance was tested by Levene’s test. The differences among groups with homogeneity of variance were analyzed by one-way ANOVA; otherwise, they were analyzed using the Brown-Forsythe test. The differences between control groups and experimental groups were analyzed using the LSD test (homogenic variance) or the Dunnetts T3test (heterogenic variance), α = 0.05.

## 3. Results

### 3.1. General Observations and Calculation of Cerebral Organ Coefficients in Mice

**Figure 1:**
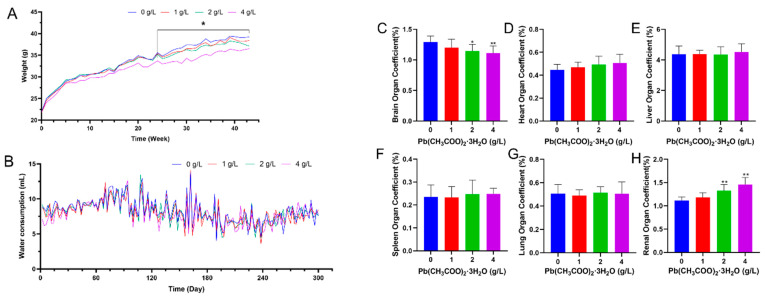
(**A**) Changes in body weight of mice in dose groups. Data are presented as the means ± SDs, * p < 0.05, *n* = 13. (**B**) Changes in water intake of mice in each dose group. Data are presented as the means ± SDs, *p* > 0.05, *n* = 13. (**C**–**H**) Effects on organ coefficients of each group of mice after 10 months of Pb exposure. The data shown are expressed as the mean ± SD. * *p* < 0.05, ** *p* < 0.01, *n* = 10.

The weight gain of mice in the high-dose group was lower than that of the other groups at the end of 10 months of Pb poisoning, especially at any of the weight monitoring points at the stage of 24 to 43 weeks of Pb poisoning, and the weight of mice in the high-dose group was significantly lower than that of the control group, with a statistically significant difference (Figure 1A, *p* < 0.05). It can be seen that the rate of weight gain of mice slowed down with the increase in the time of poison staining. The results of water intake showed that the difference in total water intake of mice between dose groups was not statistically significant (Figure 1B; *p* > 0.05).

Following the end of the treatment regimen, mice were sacrificed, and brains were weighed. Organ coefficients were calculated according to the following formula: Organ Coefficient = organ weight/body weight × 100%. Brain organ coefficients of mice gradually decreased with increasing doses of Pb exposure (Figure 1C; *p* < 0.05) in the 2 and 4 g/L Pb-exposed groups compared with the control group. It suggests that Pb exposure may have led to atrophy or degenerative lesions in the mouse brain. There was no statistically significant difference in the coefficients of the heart, liver, spleen, and lungs of mice in each dose group (Figure 1D–G; *p* > 0.05). The organ coefficient of the kidney increases with the increase in Pb exposure dose (Figure 1H; *p* < 0.05).

### 3.2. Pb Exposure Impairs Learning and Memory Abilities in Adult Mice

**Figure 2:**
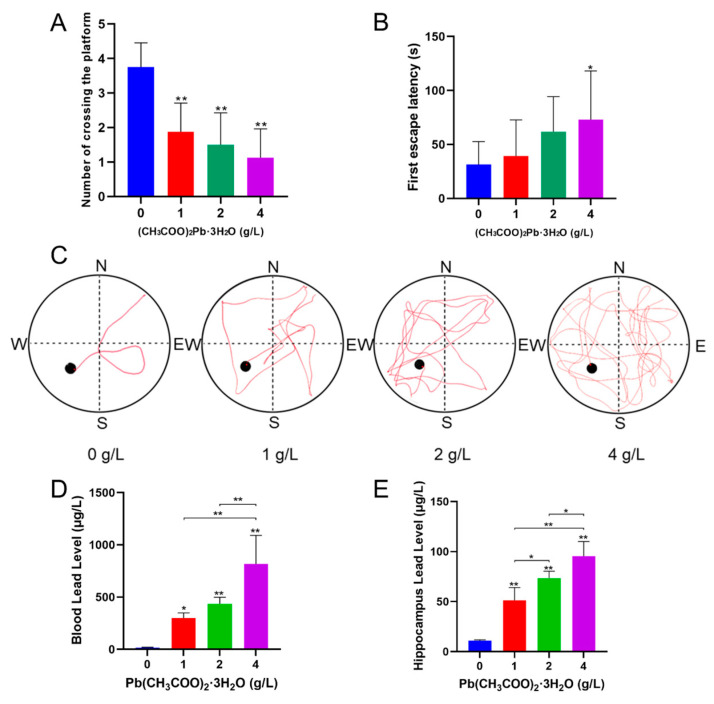
Pb dose-dependently decreases platform crossing in the Morris water maze. (**A**) Effect of exposure to different Pb concentrations on the number of crossing the platform in each group of mice after 10 months of Pb exposure. The data shown are expressed as the mean ± SEM. ** *p* < 0.01, *n* = 8. (**B**) Effect of exposure to different Pb concentrations on the first escape latency in each group of mice after 10 months of Pb exposure. The data shown are expressed as the mean ± SEM. * *p* < 0.05, *n* = 8. (**C**) Diagram of the swimming trajectory of each group of mice during the navigation test phase. (**D**,**E**) Blood Pb and hippocampal Pb levels in each group of mice after 10 months of Pb exposure. The data shown are expressed as the mean ± SD. * *p* < 0.05, ** *p* < 0.01, *n* = 6.

The MWM test was used to detect the learning and memory ability of mice. In our experiments, it was found that the number of crossing the platform was significantly lower in the remaining groups of mice with increasing doses of Pb exposure compared with the control group (Figure 2A, *p* < 0.01); the latency period of the first escape gradually increased, and the difference was statistically significant in the 4 g/L Pb-exposed group (Figure 2B, *p* < 0.05). During the hidden platform phase, the control mice clearly knew the quadrant and location of the platform. On the contrary, the mice in the Pb-exposed group had confusing swimming trajectories and could not correctly locate the quadrant and position of the platform. The degree of confusion of the swimming trajectory was elevated with the increase in the dosage of toxicity. As shown in Table 2, with the increase in the number of training days, the mean avoidance latency of mice in each dose-exposed group showed an overall decreasing trend, and on the 5th day of training, the difference between the average avoidance latency of mice in the medium and high dose groups was statistically significant compared with that of the control group (Table 2; *p* < 0.05).

Moreover, the blood Pb and hippocampal Pb serve as additional important indicators of learning and memory impairment in mice. The blood Pb and hippocampal Pb levels of mice in the Pb-exposed group were higher than those in the control group, and the differences were statistically significant (Figure 2D,E, *p* < 0.05). Apart from that, the results also showed a positive dose-response relationship.

### 3.3. Pb Exposure Disrupts the Morphological Structure of Neuronal Cells in the Hippocampus of Adult Mice

**Figure 3:**
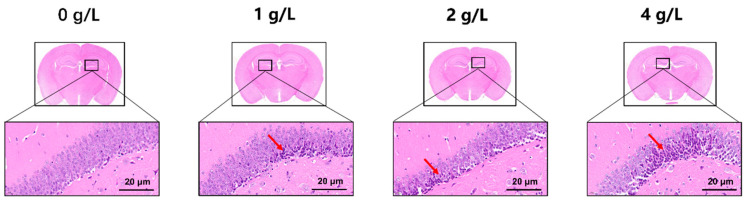
Pb exposure disrupts the morphological structure of neuronal cells in the hippocampus of adult mice. HE staining results of hippocampal tissues after 10 months of Pb exposure. Red arrow: Morphostructural damage to mouse hippocampal tissue cells. Scale bar: 20 μm.

To observe the damage of the hippocampal region in mice exposed to Pb, HE and immunofluorescence staining of hippocampal tissues were performed 10 months after Pb exposure. Hippocampal neurons of mice in the control group were arranged in a tight and orderly manner with normal cell morphology, light blue nuclei, abundant cytoplasm, and clear demarcation (Figure 3). After the exposure to Pb, some of the cells showed concentrated cytoplasm and deep-stained nuclei, mostly polygonal or shuttle-shaped (red arrow), and the abnormalities of neurons were evident with the increase in Pb exposure dose (Figure 3).

### 3.4. Pb Exposure Promotes the Aggregation of *Aβ* and p-tau in the Hippocampus

**Figure 4:**
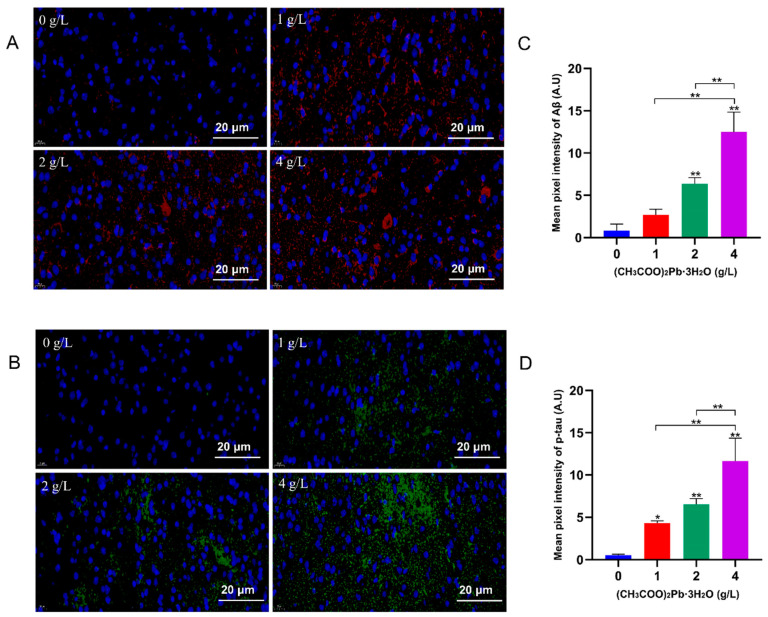
Pb exposure promotes the aggregation of Aβ and p-tau in the hippocampus. (**A**,**B**) Immunofluorescence staining results of hippocampal tissues of each group of mice exposed to Pb for 10 months (*n* = 3). Red fluorescence: Aβ; Green fluorescence: p-tau. Scale bar: 20 μm. (**C**,**D**) Mean immunofluorescence intensity of Aβ and p-tau in the hippocampus region of mouse brain after 10 months of Pb exposure. The results are expressed as *x* ± SD, *n* = 3, *: *p* < 0.05, **: *p* < 0.01.

Immunofluorescence staining indicated there was little natural deposition of Aβ and p-tau in the hippocampal region of control mice with more Aβ and p-tau accumulation in the hippocampal region of the Pb-exposed mice (Figure 4A,B). Fluorescence intensity tended to increase with the increase in dose and time of Pb exposure (Figure 4C,D).

### 3.5. Pb promotes the Expression of AD-Related Genes and Proteins

**Figure 5:**
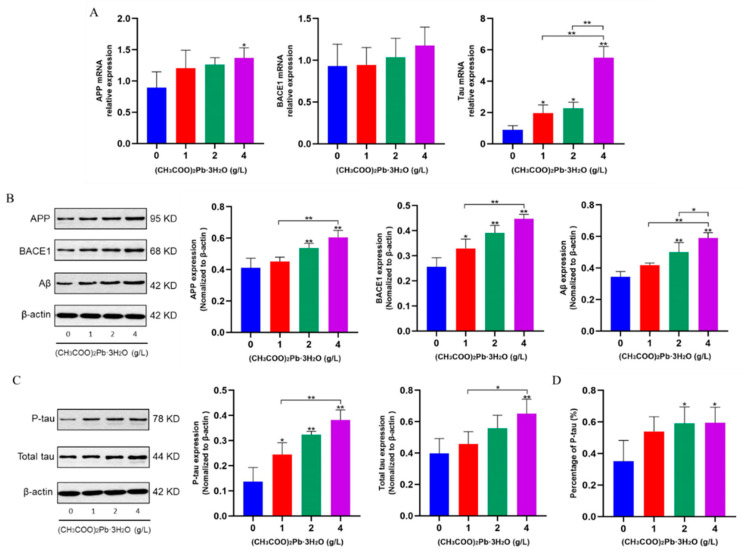
Expression levels of AD-related genes and proteins after 10 months of Pb exposure. (**A**) qRT-PCR results of AD-related genes after 10 months of Pb exposure. (**B**,**C**) Western blot results of AD-related proteins after 10 months of Pb exposure. (**D**) Phosphorylated tau protein percentage of total tau protein after 10 months of Pb exposure. The data shown are expressed as the mean ± SD. * *p* < 0.05, ** *p* < 0.01, *n* = 4.

As shown in Figure 5A–C, expression levels of genes and proteins typical of AD, such as APP, BACE1, Aβ, and p-tau, were elevated in both phases of the exposure cycle, especially in the high-dose group (Figure 5A–C; *p* < 0.01). In addition, the proportion of p-tau was calculated according to tau protein phosphorylation degree = relative expression of p-tau protein/relative expression of total tau protein × 100%. The proportion of p-tau protein gradually increased under different doses of Pb exposure, and the difference between the 2 and 4 g/L Pb-exposed groups was statistically significant compared with the control group (Figure 5D; *p* < 0.05).

### 3.6. Pb Exposure Promotes the Nuclear Translocation Process of NF-κB to Increase the Expression Levels of *Aβ* and p-tau Mediated by Downregulation of CDR1as

**Figure 6:**
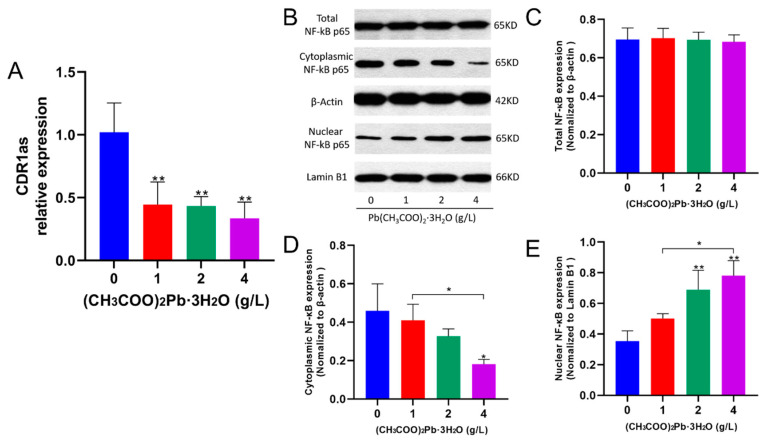
Pb exposure promotes the nuclear translocation process of NF-κB to increase the expression levels of Aβ and p-tau mediated by downregulation of CDR1as. (**A**) Expression levels of CDR1as in the hippocampus of mice in each group after 10 months of Pb exposure. The data shown are expressed as the mean ± SD, ** *p* < 0.01, *n* = 4. (**B**–**E**) Results of nucleoplasmic segregation of NF-κB in each group of mice after 10 months of Pb exposure. The data shown are expressed as the mean ± SD, * *p* < 0.05, ** *p* < 0.01, *n* = 4.

After 10 months of Pb exposure, the expression level of CDR1as in the hippocampus of each Pb-exposed group of mice was significantly reduced (Figure 6A; *p* < 0.01).

The expression level of total NF-κB protein was not different between mice in each dose group (*p* > 0.05). We performed nucleoplasmic separation of hippocampal neuronal cells and examined the expression levels of NF-κB in the cytoplasm and nucleus (Figure 6B) and found that NF-κB levels in the cytoplasm gradually decreased with the increasing dose of Pb exposure, while NF-κB levels in the nucleus gradually increased, especially in the high-dose group (Figure 6B,D,E; *p* < 0.05).

### 3.7. Pb Exposure Promotes miR-671 Expression to Induce Cleavage of CDR1as by AGO2

**Figure 7:**
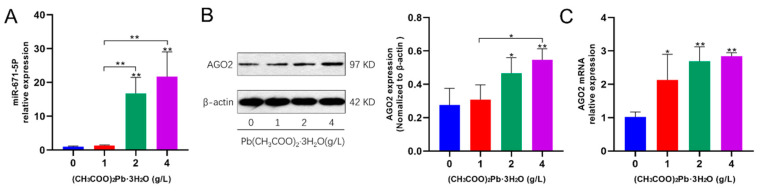
Pb exposure promotes miR-671 expression to induce cleavage of CDR1as by AGO2. (**A**) Expression levels of miR-671-5P in the hippocampus of mice in each group after 10 months of Pb exposure. The data shown are expressed as the mean ± SD, ** *p* < 0.01, *n* = 4. (**B**,**C**) Expression levels of AGO2 gene and protein in the hippocampus of mice in each group after 10 months of Pb exposure. The data shown are expressed as the mean ± SD, * *p* < 0.05, ** *p* < 0.01, *n* = 4.

To further explore the possible causes of reduced CDR1as, we also examined the expression of miR-671 in hippocampal tissues of mice exposed to Pb for 10 months. After 10 months of Pb exposure, the expression level of miR-671 increased with the dose of Pb exposure compared and was statistically significant in the medium- and high-dose groups (Figure 7A; *p* < 0.01). Next, we examined the expression levels of the AGO2 gene and protein. Expression levels of AGO2 gene and protein were elevated with increasing dose and time of Pb exposure, particularly in the 2 and 4 g/L Pb-exposed groups (Figure 7B,C; *p* < 0.05). These results suggest that the decrease in CDR1as may be the result of miR-671-induced cleavage by AGO2.

## 4. Discussion

The results from this study demonstrated that mice were exposed to Pb for most of their life cycle to construct a mouse model of AD-like lesions, which is in line with the disease spectrum characteristics of Pb as an accumulated poison leading to the development of AD. Based on the successful establishment of a mouse model of AD-like lesions caused by chronic Pb exposure, this study further explored the possible upstream and downstream pathways and modes of action of CDR1as regulation. There are still many limitations in this study. The set mode of exposure was gastrointestinal, but the exposure mode of practitioners in the actual workplace was mainly respiratory intake. Lack of specific indicators to observe the progression of AD in the course of long-term intoxication, as well as failure to observe the dynamic trend of CDR1as in the course of chronic intoxication.

**Figure 8:**
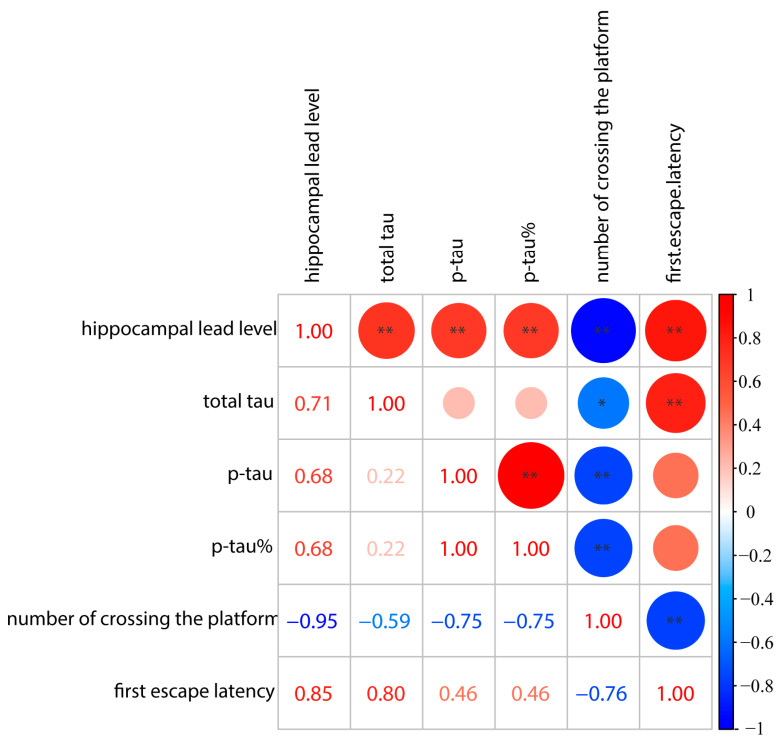
Pearson correlation analysis of hippocampal lead levels, tau protein metrics, and behavioral metrics with lead exposure after 10 months. The color and size of the circle denotes the magnitude and direction of the relationship. * *p* < 0.05, ** *p* < 0.01, *n* = 4.

A growing amount of evidence has confirmed that Pb causes multiple system disorders, with CNS being the top target in acute or chronic Pb exposure. Epidemiological evidence indicates that low-dose Pb exposure for a long period can cause a decline in spatial learning and memory abilities, especially in high-exposed occupational groups [2]. Blood Pb level is an important indicator reflecting the degree of damage and for clinical diagnosis of Pb poisoning. It was found that blood Pb levels of 1.98 μg/dL to 2.02 μg/dL in mice were associated with neurobehavioral abnormalities, such as reduced olfactory recognition and exploratory activity, decreased memory, learning ability, and intelligence [27,28]. In our study, the blood and hippocampal Pb levels in the Pb-exposed group were significantly higher than those in the control group, especially in the 4 g/L Pb-exposed group, which showed an order-of-magnitude increase in blood and hippocampal Pb levels, suggesting that the mice in the Pb-exposed group were undergoing a higher Pb-loading state and suffering from Pb-neurotoxicity damage. It was also found that mice in the Pb-exposed group were less active compared to the control group, which had previously shown the same behavior after Pb exposure for 3 months, suggesting that the effect became more pronounced with increasing concentration and time. In another in vivo study, the spatial learning and memory abilities of mice were tested by neurobehavioral tests after being exposed to different concentrations of gradient Pb-acetate solutions, and the results showed that the average escape latency of mice was prolonged and the number of times they crossed the platform position was reduced, suggesting that Pb exposure significantly impaired the learning and memory functions of mice [29,30]. In this study, the MWM test revealed that the mice in the Pb-exposed group swam chaotically and purposelessly and were unable to accurately locate the quadrant and position of the platform, and the average latency to escape was prolonged, which indicated that the mice’s spatial localization and learning ability was impaired; the number of times the mice traversed the platform and the time of the first time they reached the platform were higher than those of the control group after the platform was withdrawn, which indicated that their memory was impaired by the Pb, which was in line with the results of the previous studies [28,30]. Combined with the results of the Morris water maze experiment and the measurements of blood and hippocampal Pb levels, the mice in the Pb-exposed group showed a decrease in learning and memory ability, which was positively correlated with blood Pb levels and hippocampal Pb levels, consistent with our previous study (as shown in Figure 8).

The hippocampus is an important anatomical structure and nerve center for learning and memory capabilities, and any form of damage can Pb to learning and memory dysfunction [31,32]. The hippocampus is also a main site of damage seen in AD patients [33]. Both in the present study and in previous studies using mice exposed to Pb for three months, it was shown that the morphological structure of neuronal cells in the hippocampal region was damaged, with a large amount of abnormally increased Aβ and p-tau deposited in the hippocampal region, which would impair normal hippocampal function. Aβ and p-tau proteins are typical molecular biomarkers in the brains of AD patients and are important indicators for the clinical diagnosis of AD [34]. Therefore, in the present study, the expression levels of Aβ and p-tau proteins were detected in the hippocampal tissues of the mouse brain by Western blot, and the results showed that the Aβ and p-tau protein content increased with the increase in Pb-exposed dose. Subsequently, the expression levels of gene transcripts and proteins that are upstream indicators of Aβ and p-tau proteins, such as APP, BACE1, and tau, were also detected, and the results showed the same trend of changes as Aβ and p-tau, which is consistent with the results of previous studies [35]. These results suggest that chronic Pb exposure damages the structure of neuronal cells in the hippocampal tissue of mice and up-regulates the expression of AD-related gene transcripts and proteins, especially the increase in the expression levels of Aβ and p-tau proteins, which leads to chronic damage to the neuronal cells in the hippocampal tissue of mice. These results seem to correlate with the results of behavioral tests (Figure 8). It suggested that the impairment of learning and memory capacities in adult mice under chronic Pb exposure conditions is in a progressive state. Also, combined with neurobehavioral and pathological results, it appears that this impairment may be irreversible by the late stage of injury.

Based on the testing of population samples, the researchers found that the expression level of CDR1as in the CA1 region of the hippocampal tissue of the brains of AD patients was significantly lower compared with that of the control group. The study also suggests that CDR1as could be used as an early biomarker for the diagnosis of AD based on the results of the assay and CDR1as’s special ring structure [36]. In experimental animal studies, it has been noted that down-regulation of CDR1as in brain tissues is linked to up-regulation of miR-671 and may be associated with neurobehavioral abnormalities in animals, such as impaired learning memory and impaired PPIs, whereas this possible regulatory relationship has not been found in non-brain tissues [19]. In our previous study, CDR1as expression in the hippocampus of each Pb-exposed group of mice was slightly reduced compared with the control group after 3 months of Pb exposure, yet the difference between the groups was not statistically significant. However, after 10 months of Pb exposure, the expression level of CDR1as in the hippocampus of each Pb-exposed group of mice was significantly reduced (*p* < 0.01). These results suggest that dysregulation of CDR1as regulatory expression may be part of the etiology of AD development and that its functioning is tissue-specific.

Human CDR1as is located mainly in the cytoplasm and has more than 70 binding sites to miR-7 [14,15,37]. MiR-7 is involved in the regulation of genes in the brain [38,39,40]. The binding of miR-7 to CDR1as has been found in cell lines, and thus CDR1as is thought to exert a sponge effect on miR-7 by reducing the number of freely available miR-7 molecules [15]. MiR-7 binding sites are only partially complementary to CDR1as, ensuring that CDR1as is not cleaved by AGO2 bound to the miR-7:CDR1as complex. CDR1as also has a binding site for miR-671. In contrast with the binding site for miR-7, this binding site is almost completely complementary to miR-671, and thus miR-671 may use this binding site to mediate the slicing of CDR1as, potentially releasing its miR-7 carrier [41]. In our study, we found a significant decrease in CDR1as expression levels and an increase in miR-671 expression levels in the Pb-exposed group of mice under long-term Pb exposure conditions with increasing duration of toxicity, which we hypothesize may be caused by Pb exposure promoting miR-671 expression to induce cleavage of CDR1as by AGO2. The downregulation of CDR1as due to Pb exposure implies a possible upregulation of miR-7 as it dissociates from CDR1as.

Accumulating evidence suggests that Pb exposure can induce NF-κB hyperactivation, which promotes overexpression of APP and BACE1 and aberrant tau protein hyperphosphorylation, thereby accelerating learning and memory deficits in human and animal models [42,43]. Our previous 3-month Pb exposure study in mice revealed a dose-dependent decrease in the expression of total NF-κB. In this study, total NF-κB protein expression levels showed no difference between mice in each group. Interestingly, previous in vitro studies have proposed that CDR1as may act as an upstream regulator of NF-κB, facilitating its nuclear translocation and subsequent activation of downstream gene expression, including AD-related genes [44]. However, a lack of corresponding in vivo experimental evidence has hindered the validation of this hypothesis. This study showed that chronic Pb exposure promoted the migration of NF-κB proteins in the cytoplasm to the nucleus, which is consistent with the results of our previous study in which mice were Pb-exposed for 3 months. These results suggested a potential interaction between CDR1as and NF-κB in the context of Pb exposure, which warrants further investigation. Focusing solely on the total levels of NF-κB may not provide a complete picture of its regulatory roles in the cell, as the transcriptional activity of NF-κB is primarily determined by its nuclear localization.

Although our study revealed the effects of lead exposure on the expression levels of CDR1as and miR-671 in the hippocampal region of mice and preliminarily investigated the molecular mechanisms that may be involved in these changes, there are still some limitations of the present study that need to be addressed in future studies. First, the cerebral cortex, a crucial brain area in AD research, was not analyzed. Future research should broaden the sample scope to encompass the cerebral cortex, thereby providing a more comprehensive evaluation of Pb’s effects on various brain regions. In addition, direct measurements of miR-7 levels were not feasible in this study due to experimental and temporal constraints, which limits our understanding of the dynamic interactions between miR-7, CDR1as, and miR-671. In future studies, we will endeavor to overcome these limitations by directly measuring miR-7 levels and the transcript and protein levels of its target genes (e.g., RelA, NLRP3/Nalp3, etc.) to explore more deeply the regulatory mechanisms of these genes in response to lead exposure. Second, while a hypothesis has been posed suggesting that CDR1as might release miR-7 via an AGO2-mediated cleavage process, current conditions and time limitations precluded direct validation through RNA immunoprecipitation (RIP) or Ago2-KO cell experiments. Future investigations will focus on elucidating the precise role of Ago2 in modulating CDR1as under conditions of Pb exposure. Finally, pathological analyses indicate that future research should delve deeper into the effects of lead exposure on hippocampal pathological alterations, specifically addressing neuronal loss, glial cell activation, and potential inflammatory reactions. This approach will aid in comprehensively understanding the consequences of Pb exposure on the CNS and foster innovative prevention and treatment strategies for related ailments.

## 5. Conclusions

Based on the data, Pb inhibited CDR1as expression, and this inhibition may occur by promoting elevated expression levels of miR-671 and AGO2 proteins, and promoting the binding of miR-671 to CDR1as to induce degradation of CDR1as by AGO2 proteins, which in turn promoted the nuclear migration process of NF-κB protein, and ultimately induced elevated expression levels of AD-related proteins, impairing the learning and memory abilities of mice, and thus participating in the pathogenesis of AD. Long-term in vivo experiments with Pb exposure revealed that miR-671/CDR1as regulation and NF-κB signaling pathway may play an important role in the onset and development of AD in adult mice due to Pb exposure, but the related mechanistic pathways are still not clearly elucidated. Therefore, in future studies, we plan to construct transgenic mice and incorporate cellular models to further explore the interrelationships and interaction patterns between upstream regulators and downstream effectors centered on CDR1as.

## Figures and Tables

**Table 1:** Primer sequences information.

Gene	Primer Sequences (5′ to 3′)
CDR1as	F: TTCTGGTGTCTGCCGTATCC
	R: GTTGCTGGAAGACCTGGAGAT
miR-671-5p	F: GGTTCTCAGGGCTCCACC
	R: CCTGGAGGGGCTGGAGTTT
AGO2	F: AGCAGCACCGACAGGAGAT
	R: GACTTGTAGAACTGAATGAGCAACT
NF-κB	F: AGAGAAGCACAGATACCACCAAGA
	R: GGTCAGCCTCATAGTAGCCATCC
BACE1	F: CAGTCCTTCCGCATCACCAT
	R: TGTTGTAGCCACAGTCTTCCAT
Tau	F: ATTACACTCTGCTCCAAGACCAA
	R: GGTTCCTCCGCTCCATCATC
APP	F: GCAGCGAGAAGAGCACTAACT
	R: ATCCTCCACATCCTCATCATCATC
GAPDH	F: AGGTCGGTGTGAACGGATTTG
	R: TGTAGACCATGTAGTTGAGGTCA
β-actin	F: GCCTTCCTTCTTGGGTAT
	R: GGCATAGAGGTCTTTACGG
U6	F: CTCGCTTCGGCAGCACA
	R: AACGCTTCACGAATTTGCGT

**Table 2:** Mean escape latency in mice after Pb exposure for 10 months ($\bar{x}\pm S$D).

Training Days	Mean Avoidance Latency (s)			
	Group 0 g/L	Group 1 g/L	Group 2 g/L	Group 4 g/L
Day 1	67.76 ± 16.24	62.47 ± 21.71	69.03 ± 15.56	68.16 ± 16.09
Day 2	51.85 ± 14.84	61.56 ± 13.67	62.71 ± 15.61	55.12 ± 13.50
Day 3	35.51 ± 16.63	39.69 ± 13.63	41.59 ± 15.07	38.61 ± 9.26
Day 4	32.55 ± 8.17	35.86 ± 8.35	37.79 ± 13.82	43.01 ± 13.18
Day 5	15.41 ± 8.26	22.69 ± 6.93	28.69 ± 8.52 *	40.05 ± 12.47 *

*n* = 8, vs. 0 g/L, * *p* < 0.05.

## Data Availability

Data will be made available on request.
